# The compensation effect of competence frustration and its behavioral manifestations

**DOI:** 10.1002/pchj.746

**Published:** 2024-03-26

**Authors:** Liang Meng, Linglan He, Mingming Chen, Yueting Huang

**Affiliations:** ^1^ Key Laboratory of Brain‐Machine Intelligence for Information Behavior (Ministry of Education and Shanghai) School of Business and Management, Shanghai International Studies University Shanghai China; ^2^ Institute of Organizational Behavior and Organizational Neuroscience, Shanghai International Studies University Shanghai China; ^3^ College of Foreign Languages, Sichuan University Chengdu China

**Keywords:** compensation effect, competence frustration, meaning maintenance model, need restoration, self‐determination theory

## Abstract

The frustration of competence, one of the three basic psychological needs proposed by self‐determination theory, has been widely demonstrated to negatively influence one's motivation and well‐being in both work and life. However, research on the recovery mechanism of competence is still in the nascent stage. In this study, a two‐stage behavioral experiment was conducted to examine the restoration of competence and the potential moderating role of resilience. Results showed that individuals who were asked to recall experience of competence frustration performed better on subsequent tasks, manifesting their behavioral efforts of competence restoration. However, resilience does not play a significant moderating role in competence restoration. Through convergent behavioral evidence, findings of this study demonstrate the compensation effect of competence frustration.

## INTRODUCTION

According to the self‐determination theory (SDT), human beings have three basic psychological needs, which are competence, autonomy, and relatedness (Ryan & Deci, 2000). As consistently shown by previous empirical studies, satisfaction of these needs can promote intrinsic motivation, improve work performance, and foster a sense of well‐being (Deci et al., 2017; Van den Broeck et al., 2016), which highlights the importance of these basic needs for individuals. In the workplace, however, need frustration, especially competence frustration, is ubiquitous. Excessive work challenges, difficult tasks, and negative feedback can lead to the frustration of employees' competence (Ryan & Deci, 2017) and further bring about negative outcomes, such as burnout, stress, depression, and anxiety (Bartholomew et al., 2014; Rouse et al., 2020). Learning about the consequences of competence frustration and the approach to deal with it is therefore a concern for psychological researchers, employees, and managers alike to address.

Previous studies have validated the negative impact of need frustration in varied areas, such as work, education, and sports (Earl et al., 2017; Gunnell et al., 2013; Warburton et al., 2020). Few studies, however, have explored need restoration after its frustration, which should not be ignored as an effective response to need frustration. In the few existing studies, scholars mainly examined the restoration of autonomy and relatedness, such that individuals who were deprived autonomy beforehand showed higher motivation to engage in following autonomy‐supporting tasks (Radel et al., 2011). In a similar manner, a pioneering study revealed the “positive” impact of competence frustration outside of the context of frustration by demonstrating that sufficiently high levels of competence frustration would activate a restoration process in which individuals desire to regain competence in subsequent tasks (Fang et al., 2017). The meaning maintenance model (MMM) provides a theoretical perspective to help understand the restoration of basic psychological needs (Meng & Ouyang, 2020). MMM proposes that humans have the fundamental need to pursue meaning and engage in compensatory behaviors to reconstruct meaning when it has been threatened (Heine et al., 2006). Importantly, compensatory behavior can occur outside of the threatened domain through fluid compensation, that is, one may seek to restore meaning in domains other than the frustrated one (Heine et al., 2006; Proulx & Inzlicht, 2012). Since competence is an important source of meaning for humans, the main purpose of this study was to examine whether individuals who had suffered competence frustration beforehand would invest more effort in subsequent tasks to restore competence, and the behavioral manifestations.

It is worth noting that in the existing research on need frustration, scholars primarily focused on its direct consequences, rarely considering the impact of individual difference factors, let alone those specific to need restoration. Resilience reflects one's ability to properly respond to multiple stressors and adverse situations (Fletcher & Sarkar, 2012; Rutter, 1987). Individuals with high resilience, relative to those with low resilience, respond more positively when faced with setbacks (Luthar et al., 2006). Recently, the first attempt was made to explore the role of resilience in need frustration, finding that resilience is a key factor that predicts one's attentional bias toward competence‐related cues after one has experienced competence frustration, and that this stimulated attentional bias contributes to recovery from competence frustration (Waterschoot et al., 2020). Based on this pioneering finding, we aimed to include resilience directly in a behavioral experimental study of competence restoration to test whether resilience as an individual difference factor would facilitate recovery from competence frustration.

Taken together, echoing the proposal of Fang et al. (2017), this study aimed to extend existing research on competence frustration by examining the behavioral manifestations of competence restoration. Specifically, whether competence frustration would result in a compensation effect and whether resilience plays a moderating role in the restoration of competence were examined by a two‐stage experiment in which the first stage manipulated competence frustration through recall of past work experience and the task in the second stage provided individuals with an opportunity for competence recovery.

## LITERATURE REVIEW AND HYPOTHESES DEVELOPMENT

### Frustration and restoration of basic psychological needs

As a mainstream theory on human motivation, SDT proposes that human beings are active organisms with a need for self‐realization and growth (Deci & Ryan, 1985). Previous research has shown that satisfaction of the basic psychological needs leads to varied positive effects (Deci et al., 2017; Eccles & Wigfield, 2002; Sparks et al., 2016), while need frustration is associated with a range of negative outcomes (Jang et al., 2016). For instance, research found that job pressure can cause employees' need for autonomy to be frustrated and lead to reduced engagement and worse performance (Bartholomew et al., 2014), while competence frustration can bring about counterproductive behaviors (Van den Broeck et al., 2014) and negatively affect life satisfaction and life quality (Trépanier et al., 2016).

Given that need frustration and negative experiences are highly correlated, individuals who experience need frustration tend not to passively accept it, but rather show a greater desire or take certain actions hoping to diminish the negative feeling and restore the frustrated need. In terms of autonomy, when individuals whose autonomy has been thwarted perform other activities that are not featured by external control, they show enhanced motivation in participation so as to regain autonomy (Fang et al., 2022; Radel et al., 2014). Similarly, individuals who have experienced relatedness frustration beforehand have a stronger desire for relatedness as a compensation (Sheldon & Gunz, 2009). In contrast, recovery from competence frustration has not received much research attention, with a few pioneering studies as exceptions (Fang et al., 2017). To illustrate, a recent study found that by reflecting on memories of prior successes, individuals who experienced competence frustration reported an increase in competence satisfaction, indicating a recovery of competence (Austin & Costabile, 2021). Electrophysiological evidence also informs the process of competence restoration, with those experiencing competence frustration in a prior highly difficult task showing greater motivation for competence restoration in performing subsequent tasks of moderate difficulty, especially those who have low performance goal orientation (Fang et al., 2019). In addition, another study explored one's desire for competence recovery as reflected by the greater attention to competence‐related cues after receiving negative feedback on a task (Waterschoot et al., 2020). Although their findings suggest that this attentional bias contributes to the recovery of competence over time, there is still a lack of direct behavioral evidence for the process of competence restoration. With this research gap, in this study, we aim to illustrate the process of competence recovery by using objective performance indicators to characterize the behavioral outcomes of one's competence restoration after its frustration.

### Meaning maintenance model and the compensation effect

Proposed by Heine et al. (2006), the MMM integrally demonstrates the innate human need for meaning and the ongoing pursuit of it. According to the MMM, human beings are constantly striving for meaning, which is the mental representation of the expected or predictable relationship that people construct to connect the self with the external world (Heine et al., 2006). When the actual experience of meaning contradicts the individual's expected meaning, it induces a strong motivation for the individual to maintain meaning (Proulx & Inzlicht, 2012). Notably, one may not respond directly to this violation, but rather attempt to reconstruct the meaning system by seeking meaning in other domains through fluid compensation (Heine et al., 2006). This theory provides generalized theoretical support for threat‐compensation studies (Proulx & Heine, 2010).

With specific reference to competence frustration, we believe that the MMM and the fluid compensation mechanism therein can provide strong theoretical support and guidance for the restoration of competence. First, the desire for competence is essentially a quest for meaning. People can construct a connection between themselves and their external environment through their perception of competence. Second, consistent with the MMM, people are natural meaning seekers, while competence frustration is a violation of the meaning framework. Such inconsistency would stimulate one's compensatory behavior. Third, according to the fluid compensation mechanism, such compensation can occur in domains where meaning is more readily available and not necessarily in the threatened domain. In addition, drawing on relevant research findings (Fang et al., 2017; Sheldon & Gunz, 2009), we suggest that competence frustration will trigger one's fluid competence recovery mechanism and the following hypothesis is proposed:

Hypothesis 1 Competence frustration would activate the mechanism of competence restoration. Specifically, a stronger motivation for competence restoration would be shown in the competence frustration group compared to the control group, resulting in higher performance in the subsequent task of moderate difficulty.

### Resilience and need restoration

Since competence frustration is generally perceived as a threatening adverse situation, personality traits for coping with this stressful state may play a fundamental role in effectively eliminating the sense of threat and restoring competence. Therefore, resilience, one's ability to cope adequately with multiple stressors (Fletcher & Sarkar, 2012), draws our attention. Resilience as a protective individual difference factor can act as a buffer against negative events. Those with high resilience, as opposed to those with low resilience, can reverse unfavorable situations and bring about better outcomes by responding constructively to stress and adversity (Hosseini & Besharat, 2010; Parsons et al., 2016). In the SDT framework, existing research has preliminarily found the predictive role of resilience on basic psychological need satisfaction and frustration (Dursun et al., 2022; González et al., 2019) and vice versa, that is, the impact of need satisfaction and frustration on resilience. For example, basic psychological need satisfaction can enhance one's resilience, which in turn results in good adaptive outcomes, such as performance (Liu & Huang, 2021), whereas the frustration of psychological needs weakens one's resilience (Trigueros et al., 2022). Waterschoot et al. (2020) first explored the role of resilience in need restoration by examining the attentional bias of highly resilient individuals to competence‐related cues after receiving negative feedback, and the subsequent impact on competence restoration. Presumably, resilience would help individuals react more positively when faced with the negative event of competence frustration, and become more motivated to recover competence, which is what the present study intends to explore. Accordingly, we propose the following hypothesis:

Hypothesis 2 Resilience promotes competence restoration. Specifically, after experiencing competence frustration individuals with higher resilience would be more motivated to recover their sense of competence by performing better in subsequent tasks.

## METHODS

### Participants

Participants who had work experience were recruited through the Mechanical Turk (MTurk) platform for this study, and were instructed to work on tasks on Qualtrics. To ensure high quality of the research data, only those employed individuals with a response approval rate of 95% or more and a response approval number of more than 100 had access to our research link. We also restricted participation of the participants who had previously completed either the memory recall task or the image emotion‐labeling task in this behavioral experiment to avoid data contamination. Given that the new paradigm of the image emotion‐labeling task was specifically developed for this study, we conducted a pilot study to optimize the presentation and procedure of the image emotion‐labeling task. Utilizing feedback from our participants, we fine‐tuned the experimental procedure to ensure clarity and prevent any potential confusion for participants in the formal experiment. Employing the G* Power software, we determined that to attain an 80% statistical power level with a medium effect size (f = 0.3) and a significance threshold of 0.05, a minimum sample size of 90 was required. The final experimental data were collected from 98 active employees from the United States and Canada, 49 (50%) of whom were male. Participants ranged in age from 22 to 66 years (*M* = 39.73 years, *SD* = 10.811 years).

### Design

This experiment has a two‐stage between‐subject design. Recall of past experiences has been widely demonstrated to influence individuals' current emotion, well‐being, and motivation (Houle & Philippe, 2017; Philippe et al., 2011). In the field of SDT, many studies have achieved effects on individuals' current state by having them recall previous experiences of need satisfaction or frustration. For instance, activating one's competence satisfaction by recalling previous competence satisfaction experiences was found to enhance their current well‐being (Philippe et al., 2012), and diminish competence frustration (Austin & Costabile, 2021). Drawing on these studies, a memory recall task was adopted in our first experimental stage to achieve manipulation of competence frustration. In the second stage, participants were asked to perform an image emotion‐labeling task of abstract paintings, which is generally adopted in behavioral experiments as a valid way to assess individual performance (Meng & Ouyang, 2020; Wang et al., 2022); it tests their motivation and effort to recover competence by measuring their performance in the task.

### Procedure

After the participants were randomly divided into competence frustration and competence satisfaction groups, they first participated in the first stage of a memory recall task in which they were asked to recall and describe, as vividly and in as much detail as possible, a competence frustration and a competence satisfaction experience, respectively. Participants in the competence frustration group were asked to recall a work experience in which they put in sufficient effort but ultimately performed poorly and failed to meet their psychological expectations or objective standards. Moreover, the activities involved in this experience should be of great importance to the participants. Since the representative sources of competence frustration in the workplace are usually highly challenging work, too difficult tasks, upward social comparison, or negative feedback (Ryan & Deci, 2017), several examples of competence frustration experiences, such as a highly difficult work assignment or a failed promotion, were given to participants.

The competence satisfaction group, on the other hand, recalled the experience of competence satisfaction at work. Compared to the competence frustration group, this experience also required sufficient effort from the participants and involved activities that were highly valued by them. The difference, however, was that the participants in the competence satisfaction group recalled a work experience that turned out to be very satisfying for them, meeting their psychological expectations and the external objective criteria. Subsequently, following completion of the memory recall task, participants in both groups were asked to report their competence frustration based on their feelings during the described experience and resilience based on general feelings in daily life.

**FIGURE 1:**
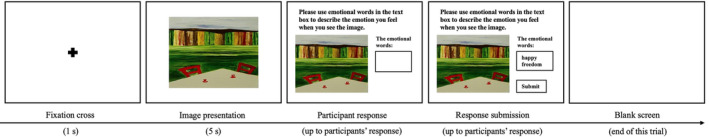
The procedure of the image emotion‐labeling task.

In the second stage of the experiment, both groups of participants were asked to complete an image emotion‐labeling task of abstract paintings. Participants were required to provide single‐word emotional tags that describe their emotions upon watching each of the 11 (one for the practice trial, and 10 for the main task) abstract paintings presented on the screen. To help participants understand the task, they first performed a practice trial before the main task. In each round, participants were first shown a fixation cross in the center of the screen for 1 s, followed by an abstract painting for 5 s, after which they were asked to label the image by typing emotional words into a text box (see Figure 1). In this study, it was emphasized several times to the participants that they should use emotional words for labeling to improve the objectivity of the final labeling quality evaluation.

The image emotion‐labeling task was moderately difficult, with no standard answers, and participants were free to choose whether or not to answer attentively according to their personal preference. Thus, this task provided an opportunity for participants in the competence frustration group to regain competence. In this task, we collected data on participants' labeling time and labeling data for each image and judged their performance in this task by the time they spent on labeling, the number of labels, and the quality of the labels provided. The higher the participants' task performance, the higher their motivation to recover their sense of competence through the compensation process and the stronger the function of the competence restoration mechanism.

### Measures

Competence frustration was measured with the widely used 4‐item Competence Frustration and Satisfaction Scale for the Work Domain (Chen et al., 2015; Fang et al., 2017). The items (e.g., “I feel disappointed with my performance in that activity”) were rated on a 7‐point Likert scale ranging from 1 (*Strongly disagree*) to 7 (*Strongly agree*). Resilience was measured by the Connor–Davidson Resilience Scale (Campbell‐Sills & Stein, 2007), which consists of 10 items, including “I always try to see the humorous side of problems.” Items were rated on a 7‐point Likert scale as well. A reliability test using SPSS 26.0 showed high internal consistency for both the competence frustration scale (α = .952) and resilience items (α = .939).

## RESULTS

### Manipulation check

We conducted an independent sample *t*‐test to test the manipulation of competence frustration of the two groups in the first stage of the experiment. Data analysis showed that competence frustration of the competence frustration group participants (*n* = 44, *M* = 5.14, *SD* = 1.31) was significantly higher than that of the competence satisfaction group participants (*n* = 54, *M* = 1.83, *SD* = 1.30; *t*(96) = 12.441, *p* < .001), indicating that this experiment successfully achieved manipulation of competence frustration.

### Measurement of task performance

This study objectively measured the performance of the image emotion‐labeling task in the second experimental stage in terms of task completion time, task completion quantity, and task completion quality. First, task completion time refers to the participant's average time spent in labeling each image. Since this study was conducted online, the problem of abnormal image labeling time may take place if participants do not concentrate on the task and stay on a single page for too long, in which case the abnormal labeling time data of the specific trial needs to be discarded from analysis. To ensure the quality of the results, we used the triple standard deviation method for each image to eliminate the abnormal time data for labeling that image, and then calculated the average labeling time for each participant respectively to obtain the final task completion time data. Second, task completion quantity is the average number of words that a participant filled in when labeling images, which was measured by calculating the average number of words labeled on each image for each participant, respectively. Third, task completion quality is the number of valid words filled in by a participant. A valid word is determined by whether it is an emotional word, such as “excited,” “calm,” or “scared.” Conversely, invalid words are non‐emotional words, such as “colored” and “abstract,” or words that only describe the content of the image. In order to make the results of valid words judgment more accurate and objective, three evaluators were invited to assess the validity of the words filled in by the participants using emotional words as the criteria, and the majority rule was followed for the few results where there was disagreement. The consistency of the assessment results among the three evaluators was 98.30%.

We examined the means and standard deviations of task completion time, task completion quantity, and task completion quality, as well as the Pearson correlations between them. The results showed that task completion time was positively correlated with both task completion quantity (*r* = 0.761, *p* < .01) and quality (*r* = 0.551, *p* < .01), and that task completion quantity and quality were also significantly positively correlated with each other (*r* = 0.756, *p* < .01; Table 1).

**TABLE 1:** Means, standard deviations and correlations of the three performance indicators.

Variables (*N* = 98)	Mean	*SD*	1	2	3
1 Task completion time	11.84	6.43	–		
2 Task completion quantity	2.47	1.17	0.761**	–	
3 Task completion quality	1.83	0.76	0.551**	0.756**	–

*Note*: Two‐sided tests.

** *p* < .01

### Data analyses

To measure the participants' performance in the second stage of the experiment, we compared task completion time, task completion quantity, and task completion quality for participants in the two groups, using independent samples *t*‐tests. First, in terms of task completion time, the difference between the average labeling time per painting spent by the competence frustration group (*M* = 13.12, *SD* = 6.85) and the competence satisfaction group (*M* = 10.80, *SD* = 5.92; *t*(96) = 1.799, *p* = .075) was marginally significant. The reason for the lack of significant difference in task completion time may be some uncontrollable confounding factors, such as participants' typing speed, network quality, and language proficiency, which largely affect participants' response time. As a result, we consider that the experimental results of task completion time are generally consistent with the first hypothesis. Second, in terms of task completion quantity, participants in the competence frustration group labeled significantly more words (*M* = 2.73, *SD* = 1.30) than those in the competence satisfaction group (*M* = 2.26, *SD* = 1.03; *t*(96) = 1.994, *p* = .049). Third, with respect to task completion quality, participants in the competence frustration group provided significantly more valid words (*M* = 2.08, *SD* = 0.71) than those in the competence satisfaction group (*M* = 1.64, *SD* = 0.75; *t*(96) = 2.957, *p* < .01). Thus, the experimental results of task completion quantity and task completion quality were in accordance with the first hypothesis of this study. In general, Hypothesis 1 was supported.

To test the moderating effect of resilience on participants' second‐stage task performance during the restoration of competence (Hypothesis 2), we first conducted a correlation analysis of resilience with average labeling time, average number of labeled words, and the number of valid labeled words, respectively. No significant correlations were found between resilience and average labeling time (*r* = 0.062, *p* = .547), average number of labeled words (*r* = 0.070, *p* = .491), or the number of valid labeled words (*r* = 0.020, *p* = .842). Then, we used Model 1 in PROCESS and set the bootstrap sample size of 5000 to test the moderating effect of resilience in the relationship between competence frustration and each of the three task performance indicators. The results showed that the interaction term between competence frustration and resilience was not significant for any of the three indicators, including average labeling time (*b* = −0.244, *p* = .441, 95% CI = [−0.869, 0.382]), average number of labeled words (*b* = −0.070, *p* = .235, 95% CI = [−0.185, 0.046]), and the number of valid labeled words (*b* = −0.059, *p* = .122, 95% CI = [−0.135, 0.016]), after controlling for the participants' age, gender, education, and ethnicity (Table 2).

**TABLE 2:** The moderation effects of resilience.

Regression (*N* = 98)		Fit index				Significance of regression coefficients			
Dependent variables	Independent variables	*R* ^2^	*F*	*p*	*b*	*SE*	95% CI	*t*	*p*
Average labeling time	Competence frustration	0.157	2.388*	.028	0.339	0.322	[−0.301, 0.980]	1.053	.295
	Resilience				0.707	0.755	[−0.794, 2.207]	0.935	.352
	Interaction				−0.244	0.315	[−0.869, 0.382]	−0.774	.441
Average number of labeled words	Competence frustration	0.139	2.077	.054	0.063	0.059	[−0.055, 0.181]	1.061	.291
	Resilience				0.130	0.139	[−0.147, 0.407]	0.932	.354
	Interaction				−0.070	0.058	[−0.185, 0.046]	−1.196	.235
Number of valid labeled words	Competence frustration	0.118	1.724	.113	0.044	0.039	[−0.033, 0.122]	1.138	.258
	Resilience				0.084	0.091	[−0.098, 0.265]	0.918	.361
	Interaction				−0.059	0.038	[−0.135, 0.016]	−1.559	.122

*Note*: Two‐sided tests. The coefficients for control variables were omitted in Table 2.

* *p* < .05.

Subsequently, to further test the effect of resilience, we performed an analysis of variance (ANOVA) with resilience as a covariate, controlling for the effect of resilience on the results. The significance levels of the differences in task performance between the two groups obtained by independent samples *t*‐test were compared with those obtained by ANOVA using resilience as a covariate. The results indicated that, after controlling for resilience, the significance levels of the between‐group differences with respect to the average labeling time, average number of labeled words, and the number of valid labeled words were slightly reduced (Table 3). Combining these results, we conclude that resilience does play a role in the recovery process of competence, but the effect is not significant. Thus, Hypothesis 2 was not supported.

**TABLE 3:** Comparison of significance levels of differences in task performance between two groups with/without the individual difference factors of resilience.

Task performance	*p*‐values of independent samples *t*‐test	*p*‐values of ANOVA between with resilience as a covariate
Average labeling time	.075	.054
Average number of labeled words	.049	.033
Number of valid labeled words	.004	.003

## DISCUSSION

Based on previous research, this study focused on the behavioral manifestation of the recovery mechanism of competence after its frustration and the role of resilience as an individual difference factor in the restoration process. By conducting a two‐stage behavioral experiment, the present study found that competence frustration would indeed activate the competence restoration process. Individuals who were induced to experience competence frustration through memory recall were found to have increased time investment, and greater quantity and quality of task completion in subsequent tasks of moderate difficulty in order to recover competence. Meanwhile, after controlling for the effect of resilience, the difference in performance in subsequent tasks between the competence frustration and competence satisfaction groups was more pronounced. Nevertheless, the significant facilitation role of resilience in the competence restoration process was not demonstrated.

This study innovatively proposed three behavioral indicators to measure participants' effort provision during competence recovery. The fact that the emotion‐labeling task was easy enough to most participants and did not have standard answers allowed for better competence restoration. Since there are no standard answers, the more effort they expended, the better performance they would achieve, which helps restore competence. We believe the measurement of multiple objective indicators in this study may provide inspiration for subsequent research and help enrich the approach to measure the competence recovery process.

### Theoretical implications

Competence is one of the three basic psychological needs of human beings that affect their psychological growth, well‐being, and performance (Bartholomew et al., 2014; Ryan & Deci, 2000). Competence frustration has been found to be negatively related to individuals' motivation and behavior in school, life, and work (Earl et al., 2017; Rouse et al., 2020; Warburton et al., 2020). However, there is a dearth of research on the recovery mechanism of competence. This study explored competence restoration after its frustration based on the fluid compensation effect proposed in the meaning maintenance model. Overall, individuals who suffered from competence frustration in the first stage of the memory recall task had higher performance in the second stage of the image emotion‐labeling task. Thus, this study is the first to manifest competence restoration on the observable behavioral level.

In contrast to previous studies in which participants experienced competence frustration directly from a specific task (Fang et al., 2017, 2019; Waterschoot et al., 2020), this study used autobiographical memory to evoke competence frustration that participants had experienced before, and behaviorally illustrated the compensation effect following such competence frustration. On one hand, this suggests that competence frustration experiences can have a long‐lasting impact on individuals, and that the compensation effect can be triggered by mere recall. On the other hand, in line with previous findings (Fang et al., 2017), we verify that the recall of competence frustration as a negative experience may also have a positive side, as it can motivate individuals to be more active and engaged in the current task by stimulating compensatory behaviors.

In addition, previous studies on competence restoration have primarily drawn on one's motivation level to reflect the competence recovery process, neglecting to capture the behaviors that help restore competence and the outcomes. For example, Waterschoot et al. (2020) characterized one's intention to recover competence through the attentional bias to competence‐related cues, and Fang et al. (2017) showed competence recovery through one's higher self‐reported intrinsic motivation in subsequent tasks. While illuminating, these studies did not include specific indicators to objectively reveal the process of competence restoration. In this study, based on the MMM, the behavioral experimental method was adopted to measure participants’ performance through three objective dimensions, namely, task completion time, and task completion quantity and quality in the image emotion‐labeling task, which behaviorally reflects the recovery mechanism after competence frustration and fills the research gap of previous studies lacking behavioral indicators of competence restoration. Measuring individuals' competence restoration at the behavioral outcome level not only improves the objectivity and validity of the findings, but also provides important references for subsequent competence restoration studies.

The MMM emphasizes humans’ persistent pursuit of meaning, and the fluid compensation effect provides theoretical support for the finding that individuals who are frustrated in one domain would desire to recover meaning in other domains (Heine et al., 2006). While previous studies have explained fluid compensation in the context of self‐esteem, certainty needs, belonging needs, and meaning disappearance (Heine et al., 2006; Meng & Ouyang, 2020), the present study expands the application contexts of fluid compensation in terms of the recovery mechanism after competence frustration and again validates the robust role of fluid compensation in threat‐induced compensation situations.

### Practical implications

Since the sources of competence frustration and the indicators of competence restoration in this study are highly consistent with real management contexts, this study can provide some insights relevant to managerial practices. First, employees and managers alike should raise awareness of competence frustration and recognize that it is so prevalent in the workplace that actions should be taken to address it. Second, as far as managers are concerned, competence frustration can bring negative emotional experiences to employees and thus affect their performance (Rouse et al., 2020; Van den Broeck et al., 2014). Consequently, managers are advised to avoid imposing excessive work pressure and challenges on employees, and to assign them other tasks that are conducive to restoring competence after they experience competence frustration so as to help them maintain their physical and mental health and good performance. Just as importantly, this study revealed the powerful positive impact of competence frustration memory, which managers can appropriately use to motivate employees to be more engaged and perform better in the present. Third, for the employees themselves, they should actively perform self‐regulation when experiencing competence frustration and try to regain competence by devoting themselves to other easier tasks. Additionally, employees are encouraged to take the initiative to increase their own resilience to aid the restoration of competence.

### Limitations and future directions

Undeniably, there are still some limitations in this study. First, this experiment was conducted online, which is susceptible to some uncontrollable confounding factors compared to laboratory experiments. In future research, more rigorously designed laboratory experiments should be conducted to replicate the findings of this study.

Second, the moderating role of resilience in competence restoration was not validated in this study. On this issue, we consider that it may be due to the fact that one's participation in the image emotion‐labeling task is essentially a resource investment process. Although individuals with high resilience are less susceptible to the negative effects of setbacks (Parsons et al., 2016), engagement in a task that helps restore one's competence necessitates a continued investment of personal resources following an experience of setback. Therefore, one's performance in the image emotion‐labeling task should be considered within the context of their available personal resources and their capacity to effectively mobilize these resources. This suggests that the mechanisms at play may be more intricate than resilience alone. Nevertheless, given the important role of resilience in responding to adverse situations, future research could attempt to further explore the effect of resilience or other possible individual difference factors on competence restoration through multi‐method research designs.

Third, besides the current findings, there are many perspectives on competence recovery that warrant further exploration by researchers. For instance, future research could further explore the competence restoration mechanism by manipulating multiple sources of competence frustration. Furthermore, effects of competence frustration on other behaviors (e.g., innovation and consumption) could be examined.

## CONFLICT OF INTEREST STATEMENT

The authors declare there are no conflicts of interest.

## ETHICS STATEMENT

This study received the ethical approval from the Ethics Committee at Shanghai International Studies University and was carried out in accordance with its requirements. All participants gave a written informed consent according to the Declaration of Helsinki. All participants had normal or corrected‐to‐normal vision. None of them reported any history of psychiatric or neurological disorders.

## Data Availability

The data that support the findings of this study are available from the corresponding author, (Mingming Chen, cmming163@163.com), upon reasonable request.
